# TRPML1—Emerging Roles in Cancer

**DOI:** 10.3390/cells9122682

**Published:** 2020-12-13

**Authors:** Yiming Yang, Xingjian Zhai, Yassine El Hiani

**Affiliations:** Department of Physiology and Biophysics, Dalhousie University Faculty of Medicine, Halifax, NS B3H 4R2, Canada; Yiming.Y@dal.ca (Y.Y.); xn980835@dal.ca (X.Z.)

**Keywords:** cancer, lysosomes, TRPML1, mitochondria

## Abstract

The mucolipin-1 (TRPML1) channel maintains lysosomal ionic homeostasis and regulates autophagic flux. Defects of TRPML1 lead to lysosomal storage diseases and neurodegeneration. In this report, we discuss emerging evidence pertaining to differential regulation of TRPML1 signaling pathways in cancer progression with the goal of leveraging the oncogenic potential of TRPML1 to inspire therapeutic interventions.

## Main Document

**Figure 1:**
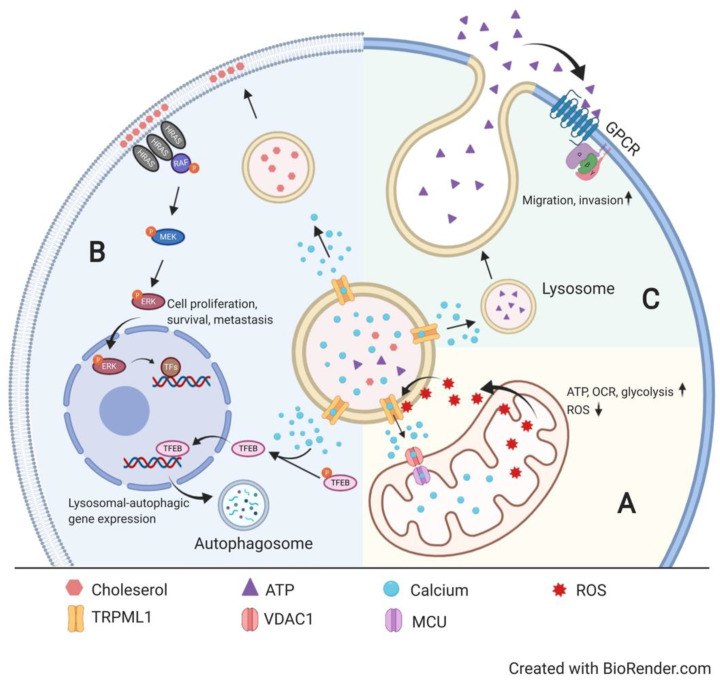
Mucolipin-1 (TRPML1) signaling in physiology and oncogenesis. (**A**) Mitochondrial reactive oxygen species (ROS) trigger TRPML1-mediated lysosomal Ca^2+^ release [9], which, in turn, facilitates mitochondrial Ca^2+^ uptake through voltage-dependent anion channel 1 (VDAC1) and mitochondrial calcium uniporter (MCU) on the outer and inner mitochondrial membrane, respectively [10]. This influx of lysosomal Ca^2+^ into the mitochondria increases mitochondrial oxygen consumption rate (OCR) and overall cellular bioenergetic output while keeping ROS production in check [11]. (**B**) TRPML1-mediated lysosomal Ca^2+^ release facilitates nuclear translocation of transcription factor EB (TFEB), stimulating transcriptional machineries of lysosomal biogenesis and autophagic flux in normal cells [9]. On the other hand, in *HRAS*-driven cancers, oncogenic TRPML1 enhances vesicular trafficking of cholesterol to the plasma membrane, enriching membrane-based proliferative signaling pathways [12]. (**C**) TRPML1 mediates extracellular release of lysosomal ATP in cancerous cells, driving tumor migration and invasion [13]. Created with BioRender.com.

The transient receptor potential (TRP) family channels were first discovered in the 1960s from behavioral mutants of *Drosophila melanogaster* displaying visual impairments to high ambient light stimulation [1]. Genetic mapping and subsequent cloning led to the identification of six subfamilies (ankyrin, canonical, melastatin, mucolipin, polycystic, and vanilloid) of mammalian TRP channels based on sequence homology [2]. TRP channels mediate a plethora of biological processes ranging from integrating neurogenic signals, such as thermosensation and nociception in sensory neurons, to modulating immune responses during inflammation and infection [3,4]. Enabling the functional versatility of TRP channels is their shared ability to mobilize and regulate the universal second messenger, Ca^2+^ [5]. A prime example of this action modality is the lysosomal Ca^2+^-releasing mucolipin-1 (TRPML1) channel, a loss-of-function mutation of which leads to type IV mucolipidosis (MLIV) [6]. A number of reviews have discussed the physiological properties and biological implications of TRPML1 [7,8]. Recently, several groups uncovered the contribution of TRPML1 in malignancy. Emerging evidence shows that, besides its canonical function in maintaining lysosomal physiology and homeostasis, TRPML1 enables lysosome–mitochondria crosstalk to modulate mitochondrial Ca^2+^ dynamics, detects and transcriptionally couples cell stress with lysosomal biogenesis, dictates membrane lipid assembly to enrich oncogenic signaling pathways, and fosters extracellular ATP release to support malignant invasion. Collectively, TRPML1 mediates stress-induced autophagy, maintains mitochondrial Ca^2+^ dynamics in non-malignant cells, while it stimulates oncogenesis by enhancing the survival, growth, invasion, and mitochondrial bioenergetic outputs of cancer cells (Figure 1).

Cells resort to autophagic degradation of cellular components to aid the synthesis of biomolecules and organelles during nutrient starvation and stress conditions [8]. The key to modulating cellular autophagy is transcription factor EB (TFEB), which, upon Ca^2+^-dependent calcineurin dephosphorylation and nuclear translocation, is a master regulator responsible for the production of lysosome-associated enzymes, promoting lysosomal biogenesis, cellular autophagy, and lysosomal clearance through orchestrating the expression of 471 genes in the coordinated lysosomal expression and regulation (CLEAR) gene network [14]. Zhang et al., through patch-clamp experiments on COS-1 cells, showed a persistent increase in TRPML1-mediated currents subsequent to oxidant exposure, proving the sensitivity of TRPML1 to oxidant challenge (Figure 1A) [9]. In addition, the group also established that the lysosomal Ca^2+^ release channel TRPML1 couples endogenous ROS sensing to the nuclear translocation of TFEB in an attempt to eliminate ROS-damaged organelles through TFEB-dependent upregulation of autophagy and lysosomal biogenesis. In the absence of TRPML1, however, autophagic induction and mitophagy recovery were both reduced subsequent to oxidant exposure. Collectively, Zhang et al. presented evidence showing molecular crosstalk among mitochondria, lysosomes, and the nucleus (Figure 1B), whereby TRPML1 creates a positive feedback loop for cells to cope with and recover from stress, resetting cellular homeostasis. Similarly, TRPML1 is also involved in the lysosomal damage response. Lysosomal rupture triggers LC3 lipidation and promotes its physical interaction with TRPML1, potentiating TRPML1-mediated calcium release and promoting nuclear translocation of TFEB to instigate the transcriptional regulation of lysosomal biogenesis [15]. Such a response can lead to the increased degradative capacity of the cell and enhanced clearance of damaged lysosomes [16,17,18]. As the physical generation and assembly of mature lysosomes originates from the formation of a cup-like structure known as a phagophore [19], it is worth noting that TRMPL1 also orchestrates autophagy induction through phosphatidylinositol 3-phosphate (PIP_3_) generation and the recruitment of PIP_3_-binding proteins to facilitate the maturation of nascent phagophores into autophagosomes, which subsequently develop into lysosomes [20]. As a result, evidence indicates that TRPML1 modulates lysosomal biogenesis and autophagy transcriptionally and via effector protein signaling, orchestrating multi-organelle stress response.

One example through which TRPML1 mediates inter-organellar crosstalk is through physically channeling lysosomes with adjacent mitochondria. Using high-resolution live-cell confocal microscopy, Peng et al. observed the functional localization of TRPML1 to the interface of mitochondria and lysosomes in regions known as mitochondria–lysosome contact sites [10]. The addition of physiological (PI(3,5)P_2_) and synthetic agonists (ML-SA1, MK6-83) of TRPML1 in wild-type HeLa cells collectively led to a sustained increase in mitochondrial Ca^2+^, while the expression of dominant-negative, non-conducting TRPML1 abolished this elevation of mitochondrial Ca^2+^. Interestingly, rising mitochondrial Ca^2+^ occurred specifically in those sharing close physical contact with lysosomes and extended mitochondria–lysosome contact tethering further stimulated both maximum and mean mitochondrial Ca^2+^. On the other hand, fibroblasts harvested from MLIV patients showed defective mitochondrial Ca^2+^ dynamics, underscoring the need for TRPML1 channels in modulating lysosome–mitochondria Ca^2+^ homeostasis in health and disease. Considering that mitochondrial Ca^2+^ dynamics are directly linked to the regulation of cell mortality [21], it stands to reason that TRPML1 may contribute to the pathogenesis of proliferative diseases.

Indeed, in the context of cancer, the TRPML1 channel seems to govern a wide range of biological processes to meet oncogenic demands. In triple-negative breast cancer (TNBC), TRPML1 has been shown to enhance extracellular ATP release to stimulate malignant invasion. It has been previously determined that metastatic MDA-MB-231 cells display elevated extracellular ATP release [22] and that TRPML1 plays a prominent role in endo-lysosomal fusion with the plasma membrane and vesicular exocytosis [23]. To concretize the link between TRPML1 and extracellular ATP exocytosis, Xu et al. observed attenuated extracellular ATP content and invasive capacity in *TRPML1*-knockdown MDA-MB-231 cells [13]. Intriguingly, the addition of extracellular ATP partially rescued the invasive capacity of *TRPML1*-knockdown MDA-MB-231 cells. In other words, both pieces of evidence point to the notion that TRPML1 enhances the efficiency of cancer cells to execute malignant invasion and dissemination by promoting lysosomal ATP release into the extracellular space (Figure 1C), making it a prominent marker for therapeutic intervention, especially in metastatic cancers, such as TNBC.

Apart from enhancing extracellular ATP release, TRPML1 regulates intracellular lipid trafficking to the cell periphery to enrich oncogenic signaling. Acting through the mitogen-activated protein kinase (MAPK) pathway and prevalently found in head, neck, and thyroid cancers, activating *HRAS* mutations enable cells to overcome replicative constraints, continuously driving tumor growth. Identifying specific therapeutic markers co-expressed with oncogenic *HRAS* is essential. Using RNAseq and quantitative polymerase chain reaction (qPCR), Jung et al. identified upregulated expression of *MCOLN1*, which encodes TRPML1, in cancer cells harboring activating (oncogenic) *HRAS* mutations and found that *HRAS* knockdown in *HRAS*-driven HN31 cells decreased *MCOLN1* expression [12]. Subsequent experiments comparing the proliferation of cancer cells bearing wild-type *HRAS* and oncogenic *HRAS* mutations revealed an intrinsic vulnerability of oncogenic *HRAS*-driven cancer cells to genetic depletion or pharmacological inhibition of TRPML1. This finding designated TRPML1 as the Achilles’ heel in conquering *HRAS*-driven cancers. Mechanistically, pharmacological inhibition of TRPML1 resulted in decreased ERK phosphorylation in *HRAS*-driven cancers only. This deficiency in ERK phosphorylation was concordant with the absence of RAS nanocluster formation at the plasma membrane following TRPML1 inhibition in oncogenic *HRAS*-driven cancer cells. A previous report established a positive correlation between H-RAS nanocluster stability and the magnitude of pro-survival MAPK signal output [24]. Jung et al., through the use of super-resolution electron microscopy (EM), further demonstrated that the lysosomal TRPML1 channel is essential for the delivery and restoration of lipid scaffolds in order for sustained membrane-bound oncogenic ERK signaling to occur. Interestingly, the exogenous delivery of cholesterol was sufficient to rescue or compensate for defective RAS nanocluster formation following TRPML1 inhibition. Collectively, this evidence supports the idea that TRPML1 downregulation in *HRAS*-driven cancers diminished endo-lysosomal cholesterol trafficking to the plasma membrane, potentially impairing the recruitment and activation of oncogenic substrates within the MAPK pathway and negatively regulating cell proliferation [25].

Because TRPML1 mediates lysosome–mitochondria Ca^2+^ transfer in non-cancerous cells, Almasi et al. examined the functional significance for such inter-organellar crosstalk in TNBC MDA-MB-231 cells [11]. *TRPML1* knockdown by two distinct shRNAs drastically reduced the viability of malignant TNBC cells but not that of the non-tumorigenic MCF-10A cells (Figure 1A). To understand underpinning pathways affected by TRPML1 depletion, targeted mass spectrometry-based metabolomics was conducted to screen for global alterations of metabolic regulators upon *TRPML1* knockdown. Alterations of metabolites in aerobic respiration were detected. In line with this finding, the overall oxygen consumption rate (OCR) of the *TRPML1*-knockdown MDA-MB-231 cells plummeted to a level equivalent to that of the non-tumorigenic MCF-10A cells. *TRPML1* knockdown, however, exerted almost no impacts on the OCR of MCF-10A cells. Concomitantly, the overall glycolytic capacity and overall ATP production were selectively impaired in cancerous MDA-MB-231 cells but not in mammary MCF-10A cells. This decrease in mitochondrial bioenergetics was accompanied by morphological changes of the mitochondria-associated membranes (MAMs) in *TRPML1*-knockdown TNBC cells. To determine the therapeutic relevance of TRPML1, Almasi et al. showed that *TRPML1* knockdown potentiated cytotoxic effects of doxorubicin on MDA-MB-231 cells, presenting TRPML1 as a potent metabolic disruptor and chemosensitizer for TNBC MDA-MB-231 cells, while sparing non-tumorigenic breast cells.

Clinically, TRPML1 has been described in an array of malignancies. In non-small-cell lung cancer, increased *TRPML1* expression is observed at high tumor stages and is positively correlated with tumor proliferation, migration, and invasion [26]. Consistent with this finding, *TRPML1* expression is also increased in pancreatic ductal adenocarcinoma (PDAC) patients and negatively correlates with patient overall survival and recurrence-free survival [27]. Since cancer cells aggressively augment protein synthesis machineries to stimulate cell proliferation, cancer cells experience elevated proteotoxic stress due to increased occurrences of translational infidelity and susceptibility to protein misfolding in the endoplasmic reticulum, potentially triggering cell death [28,29]. To avoid cell death due to proteotoxic stress, *TRPML1* overexpression in melanoma acts to tone down proteotoxic stress-inducing protein synthesis machineries driven by mTORC1 and simultaneously promotes serine homeostasis [30]. In human glioblastoma patients, on the other hand, *TRPML1* expression is decreased in comparison to that of healthy astrocytes and *TRPML1* knockdown is able to attenuate autophagic cell death upon potent ROS inducer challenge [31]. Intriguingly, TRPML2, which shares high amino acid homology with TRPML1, is upregulated in high-grade gliomas of astrocytic origin and its knockdown induces apoptotic cell death through autophagy-independent pathways, indicating the versatility of mucolipin family channels in regulating cell survival [32]. Nonetheless, it is important to appreciate the context-dependent and double-edged sword effect of TRPML1 to modulate autophagy, considering that TRPML1 senses ROS production and stimulates adaptive responses to eliminate ROS and maintain survival in some conditions, while in others, such as glioblastoma, TRPML1 activation is tumor suppressive and is associated with autophagic cancer cell death.

Despite the advancement of in silico analyses of the interactome networks of TRPML1 [33], the contribution of TRPML1 to the pathophysiology of cancer, in particular, remains largely elusive. Since TRPML1 often functions in a tetrameric assembly with other mucolipin family members, it should be of interest to determine the expression equilibrium of the various mucolipin subtypes involved in TRPML1 signaling in physiology and disease. Furthermore, because lysosomes are intricate organelles containing a complex ionic environment, the activation of TRPML1 triggers the release of lysosomal Ca^2+^ stores, which may lead to physiological responses of other endo-lysosomal ion channels in an attempt to restore ionic homeostasis. Future work may study the dynamic interactions of the TRPML1 channel with other endo-lysosomal ion channels in carcinogenesis. As TRPML1 in non-tumorigenic cells seems to mainly regulate lysosomal content, maturation, and trafficking, it is unclear as to what induces a shift of cancer cells to heavily rely on TRPML1 in oncogenesis. Identifying and intercepting molecular drivers preceding TRPML1-mediated oncogenesis may be of milestone significance.
